# Incident Comorbidities, Aging and the Risk of Stroke in 608,108 Patients with Atrial Fibrillation: A Nationwide Analysis

**DOI:** 10.3390/jcm9041234

**Published:** 2020-04-24

**Authors:** Laurent Fauchier, Alexandre Bodin, Arnaud Bisson, Julien Herbert, Pascal Spiesser, Nicolas Clementy, Dominique Babuty, Tze-Fan Chao, Gregory Y. H. Lip

**Affiliations:** 1 Service de Cardiologie, Centre Hospitalier Universitaire Trousseau et EA7505, Faculté de Médecine, Université François Rabelais, 37044 Tours, France; alexandrebodin.mail@gmail.com (A.B.); arnaud.bisson37@gmail.com (A.B.); j.herbert@chu-tours.fr (J.H.); pascal.spiesser@hotmail.fr (P.S.); nclementy@yahoo.fr (N.C.); d.babuty@chu-tours.fr (D.B.); 2 Service D’information Médicale, D’épidémiologie et D’économie de la santé, Centre Hospitalier Universitaire et EA7505, Faculté de Médecine, Université François Rabelais, 37044 Tours, France; 3 Department of Medicine, Division of Cardiology, Taipei Veterans General Hospital, Taipei 112, Taiwan; eyckeyck@gmail.com; 4 Institute of Clinical Medicine, and Cardiovascular Research Center, National Yang-Ming University, Taipei 112, Taiwan; 5 Liverpool Centre for Cardiovascular Science, University of Liverpool and Liverpool Heart & Chest Hospital, Liverpool L7 8TX, UK; gregory.lip@liverpool.ac.uk; 6 Aalborg Thrombosis Research Unit, Department of Clinical Medicine, Aalborg University, DK-9000 Aalborg, Denmark

**Keywords:** atrial fibrillation, ischemic stroke, risk evaluation

## Abstract

Background: We hypothesized that the change in stroke risk profile between baseline and follow-up may be a better predictor of ischemic stroke than the baseline stroke risk determination using the CHA_2_DS_2_-VASc score ((congestive heart failure, hypertension, age ≥75 years (doubled), diabetes, stroke/transient ischemic attack/thromboembolism (doubled), vascular disease (prior myocardial infarction, peripheral artery disease, or aortic plaque), age 65–75 years, sex category (female))). Methods: We collected information for all patients treated with atrial fibrillation (AF) in French hospitals between 2010 and 2019. We studied 608,108 patients with AF who did not have risk factors of the CHA_2_DS_2_-VASc score (except for age and sex). The predictive accuracies of baseline and follow-up CHA_2_DS_2_-VASc scores, as well as the ‘Delta CHA_2_DS_2_-VASc’ (i.e., change/difference between the baseline and follow-up CHA_2_DS_2_-VASc scores) for prediction of ischemic stroke were studied. Results: The mean CHA_2_DS_2_-VASc score at baseline was 1.7, and increased to 2.4 during follow-up of 2.2 ± 2.4 years, (median (interquartile range: IQR) 1.2 (0.1–3.8) years), resulting in a mean Delta CHA_2_DS_2_-VASc score of 0.7. Among 20,082 patients suffering ischemic stroke during follow-up, 67.1% had a Delta CHA_2_DS_2_-VASc score ≥1 while they were only 40.4% in patients without ischemic stroke. The follow-up CHA_2_DS_2_-VASc score and Delta CHA_2_DS_2_-VASc score were predictors of ischemic stroke (C-index 0.670, 95% confidence interval (CI) 0.666–0.673 and 0.637, 95%CI 0.633–0.640) and they performed better than baseline CHA_2_DS_2_-VASc score (C-index 0.612, 95%CI 0.608–0.615, *p* < 0.0001). Conclusions: Stroke risk was non-static, and many AF patients had ≥1 new stroke risk factor(s) before ischemic stroke occurred. The follow-up CHA_2_DS_2_-VASc score and its change (i.e., ‘Delta CHA_2_DS_2_-VASc’) were better predictors of ischemic stroke than relying on the baseline CHA_2_DS_2_-VASc score.

## 1. Introduction

The presence of atrial fibrillation (AF) is a recognized risk factor for ischemic stroke [1]. In these patients, the use of oral anticoagulation reduces the risk of ischemic stroke and mortality in clinical trials or observational cohorts [1].

The CHA_2_DS_2_-VASc score is calculated using the baseline risk factors for assessment of the risk of ischemic stroke risk in patients with AF [2]. However, the stroke risk in patients with AF is not static, and with time, patients get older and acquire new comorbidities. Indeed, one analysis from Taiwan reported that the change in CHA_2_DS_2_-VASc score between baseline and follow-up was a better predictor of ischemic stroke [3]. A similar observation was reported from Korea [4]. We are unaware of any similar studies in white European cohort.

We analyzed a nationwide cohort from France and we hypothesized that the change in stroke risk profile between baseline and follow-up may be a better predictor of ischemic stroke than the baseline stroke risk determination using the CHA_2_DS_2_-VASc score.

## 2. Methods

Our nationwide French longitudinal cohort study used the national database covering hospital care for the whole population. The data for all patients admitted with AF in France from January 2010 to December 2018 were collected from the national administrative database, the PMSI (Programme de Médicalisation des Systeèmes d’Information). This includes around 98% of the French population (67 million people) from birth (or immigration) to death (or emigration). As a result of this program implemented in 2004, medical activity is recorded in a database, computed, and rendered anonymous in the 1546 French healthcare facilities. Medical information includes the principal and secondary diagnoses which are identified with codes according to the International Classification of Diseases (ICD-10). Data for medical procedures were collected with the CCAM (classification commune des actes médicaux) which is the French medical reimbursement classification for clinical procedures. The PMSI contains individual pseudo anonymized information on each hospitalization that are linked to create a longitudinal record of hospital stays and diagnoses for each patient. Its reliability has been well evaluated [5] and this database has been used to study subjects with cardiovascular conditions, including those with AF or ischemic stroke [6,7,8]

This type of study was approved by our local institutional review board, on 1 December 2015 and was registered as a clinical audit. Ethical review was consequently not needed. The study was conducted retrospectively, patients were not involved in its conduct, and there was no impact on their care. Procedures for data collection and management have been approved by the Commission Nationale de l’Informatique et des Libertés (CNIL), the independent National ethical committee in France ensuring that information is kept confidential and anonymous (authorization number 1897139).

### 2.1. Study Population

**Figure 1:**
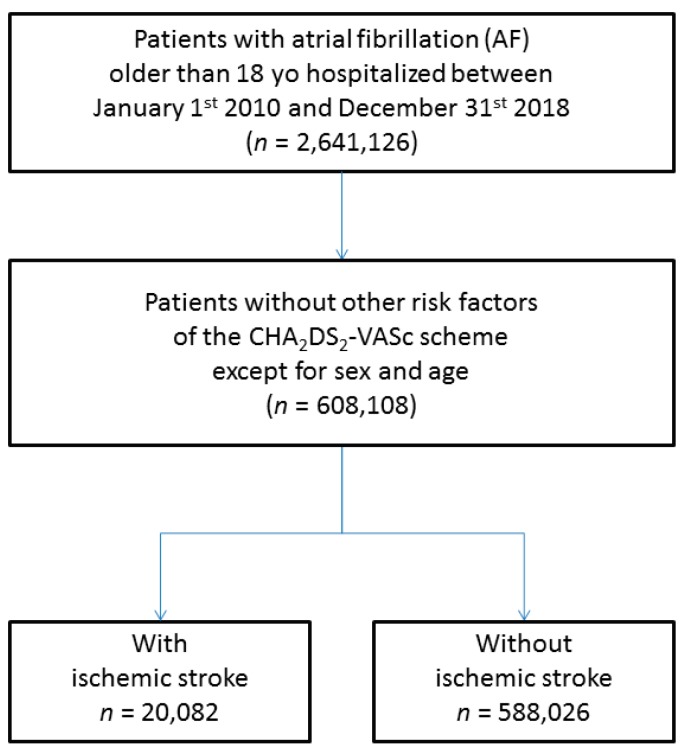
Flowchart of the study population. CHA_2_DS_2_-VASc score: (congestive heart failure, hypertension, age ≥75 years (doubled), diabetes, stroke/transient ischemic attack/thromboembolism (doubled), vascular disease (prior myocardial infarction, peripheral artery disease, or aortic plaque), age 65–75 years, sex category (female)).

We identified all patients over 18 years in the period from 1 January, 2010 to 31 December, 2018 hospitalized with a diagnosis of AF (I48 and its subsections using ICD10 codes) over the study period. The study database was constructed using the encrypted anonymized number. Patient information (including medical history and events of interest during follow-up) was described using data collected in the hospital records. Diagnoses were obtained for each hospital stay at discharge. For the present analysis, follow-up started at the date of AF diagnosis. We calculated the CHA_2_DS_2_-VASc score for every patient. In this population, there were 608,108 patients not having any risk factors of the CHA_2_DS_2_-VASc score, except for age and sex; and this was the population of interest for our study. During a follow-up of 1,322,449 person-years, 20,082 patients suffered ischemic stroke (incidence rate 1.52%/year). The flowchart of patients enrolled in the study is shown in Figure 1.

### 2.2. Baseline, Follow-Up, Delta CHA_2_DS_2_-VASc Scores, and Its Slope

The CHA_2_DS_2_-VASc scores were determined at baseline for all patients at study entry (baseline CHA_2_DS_2_-VASc score). The CHA_2_DS_2_-VASc scores at follow-up were defined as the highest recorded CHA_2_DS_2_-VASc score of each patient during the follow-up period, before the occurrence of ischemic stroke, mortality, and/or at the last news for patients free of these events. The Delta CHA_2_DS_2_-VASc score was defined as the difference between the baseline and follow-up scores (follow-up CHA_2_DS_2_-VASc minus baseline CHA_2_DS_2_-VASc scores). Among patients with a Delta CHA_2_DS_2_-VASc score ≥ 1, the rate of change in scores was also assessed by calculating the slope. The slope of CHA_2_DS_2_-VASc score change was defined as the following: log ((Delta CHA_2_DS_2_-VASc score/follow-up duration (duration in years between the index date and the date when the highest CHA_2_DS_2_-VASc score was recorded))+1).

### 2.3. Statistical Analyses

Qualitative variables are described using counts and percentages and continuous quantitative variables as means ± standard deviation and also median and quartiles when necessary. Comparisons between groups were made with chi-square tests for comparing categorical variables and the Student t test or non-parametric Kruskal–Wallis test where appropriate for continuous variables. Information on outcomes during follow-up was obtained by analyzing the PMSI codes for each patient. For the outcomes analysis, the incidence rates (%/year) was estimated in the different groups. The diagnostic accuracies of baseline, follow-up, and Delta CHA_2_DS_2_-VASc scores in predicting ischemic stroke were assessed by calculating C-indexes. The areas under the receiver-operating characteristic (ROC) curves (AUCs) of these scorings were compared using the DeLong test. Net reclassification improvement (NRI) and decision-curve analysis (DCA) were used to estimate the clinical usefulness of the predictive models. In all analyses, a p value < 0.05 was considered statistically significant. All analyses were performed using Enterprise Guide ^®^ 7.1, ^©^SAS Institute (SAS Campus Drive, Cary, NC, USA) and STATA^®^ 12, (^©^Stata Corp, College Station, TX, USA).

### 2.4. Data Access

Because this study used data from human subjects, the data and everything pertaining to the data are governed by the French Health Agencies and cannot be made available to other researchers.

## 3. Results

**Figure 2:**
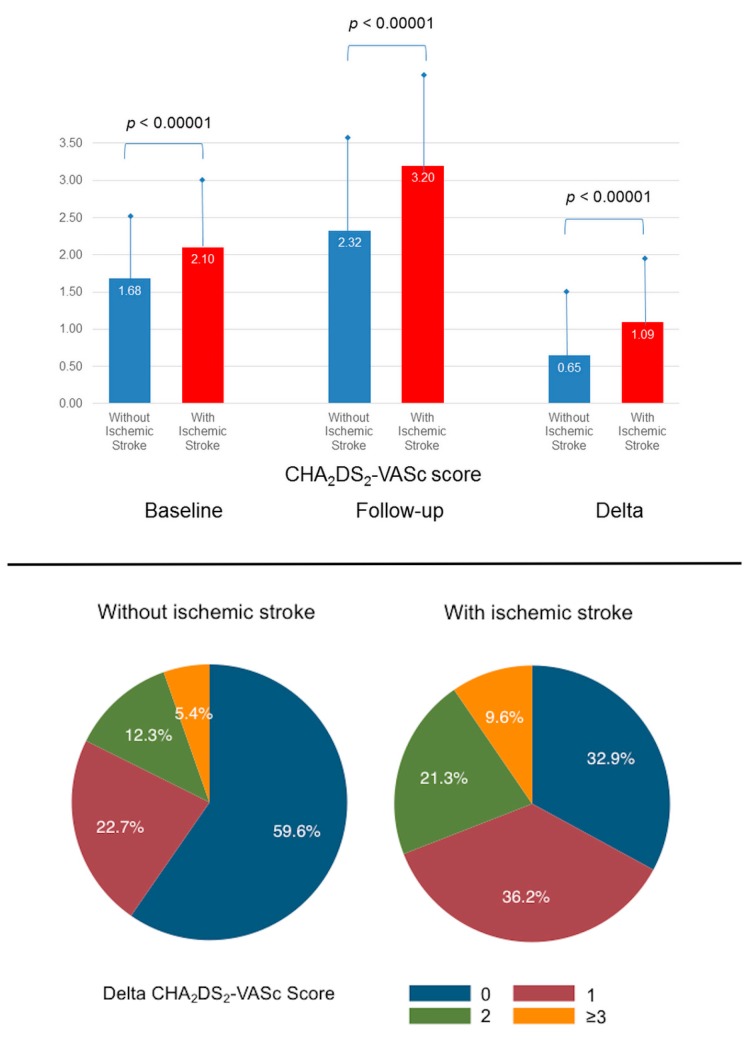
CHA_2_DS_2_-VASc scores of patients with AF with or without ischemic stroke: baseline, follow-up, and Delta CHA_2_DS_2_-VASc scores (top panel) and repartition of Delta CHA_2_DS_2_-VASc in these patients (lower panel). CHA_2_DS_2_-VASc score: (congestive heart failure, hypertension, age ≥75 years (doubled), diabetes, stroke/transient ischemic attack/thromboembolism (doubled), vascular disease (prior myocardial infarction, peripheral artery disease, or aortic plaque), age 65–75 years, sex category (female)).

The characteristics of patients with or without ischemic stroke are in Table 1. Age increased from 72.7 to 74.8 years during a mean follow-up of 2.2 ± 2.4 years (median (interquartile range: IQR) 1.2 (0.1–3.8)). The mean of the baseline CHA_2_DS_2_-VASc score was 1.7, and it increased to 2.4 during follow-up, with a mean Delta CHA_2_DS_2_-VASc score of 0.7. The baseline, follow-up and Delta CHA_2_DS_2_-VASc scores were higher for patients suffering ischemic stroke than those with no stroke (Figure 2). The % of patients with a Delta CHA_2_DS_2_-VASc score = 0 was 58.8%, meaning that more than 40% of patients had more comorbidities or became >65 or 75 years during follow-up. Around 38% of patients had ≥1 new-onset comorbidity, the most frequent being hypertension (25.7%) and heart failure (20.7%).

Among 20,082 patients who suffered ischemic stroke, 67.1% had a Delta CHA_2_DS_2_-VASc score ≥1 compared while this was the case for only 40.4% in patients with no ischemic stroke (Figure 2), and 13,041 (64.9%) patients were identified as having ≥1 new-onset comorbidity (hypertension in 9950 (49.5%), heart failure in 5889 (29.3%), diabetes mellitus in 1574 (7.8%), and vascular diseases in 3009 (15.0%)) (Table 1). In patients who experienced ischemic stroke, the number of new-onset comorbidities is shown in Supplemental Figure S1. This shows that 53.9% of patients developed 1 new comorbidity, whilst 2, 3, and 4 new comorbidities were identified in 31.8%, 11.6%, and 2.6% of patients.

### 3.1. Baseline, Follow-Up and Delta CHA_2_DS_2_-VASc Scores, and the Risk of Ischemic Stroke

The characteristics of patients by the Delta CHA_2_DS_2_-VASc score are in Table 2. The yearly risk of ischemic stroke increased continuously from 0.67% for those patients with a baseline CHA_2_DS_2_-VASc score = 0 to 2.52% for those with a baseline CHA_2_DS_2_-VASc score = 3, and from 0.60% for those with a follow-up CHA_2_DS_2_-VASc score = 0 to 1.98% for patients with a follow-up CHA_2_DS_2_-VASc score = 4 (Table 3). Considering Delta CHA_2_DS_2_-VASc score, the highest risk was seen for patients with a Delta CHA_2_DS_2_-VASc score = 1.

When considering the baseline, follow-up and delta CHA_2_DS_2_-VASc scores, using their respective score = 0 as the reference, the hazard ratios for the strata of the increasing scores are in Table 3. Increasing baseline and follow-up CHA_2_DS_2_-VASc scores were associated with a higher risk of ischemic stroke. This was also the case for subjects with delta CHA_2_DS_2_-VASc score of 1 (compared to 0 as the reference), but this association was not seen when Delta CHA_2_DS_2_-VASc score got higher.

**Figure 3:**
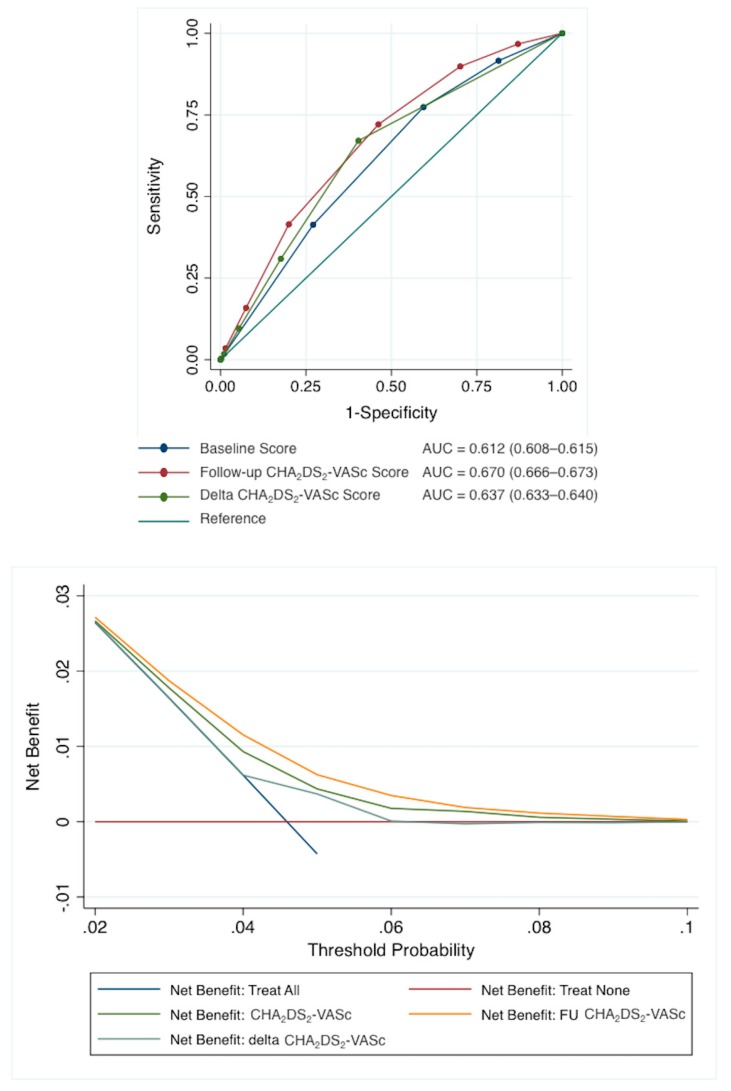
Upper panel: AUCs for the baseline follow-up and Delta CHA_2_DS_2_-VASc scores in predicting ischemic stroke. Lower panel: Decision curve analysis: net number of true positives gained with different models with different thresholds probabilities, compared to no model. AUC = area under curve

Figure 3 presents the AUC for the baseline, follow-up, and Delta CHA_2_DS_2_-VASc scores for prediction of ischemic stroke. The AUC was lower for the baseline CHA_2_DS_2_-VASc score (0.612, 95%CI 0.608–0.615) compared to that of follow-up (0.670, 95%CI 0.666–0.673) or Delta (0.637, 95%CI 633–640) scores (*p* < 0.0001 for DeLong test).

When we used NRI, we found a significant difference in the follow-up score compared to the baseline score, NRI being 14.4% (95% CI: 13.5 to 15.4%; *p* < 0.0001). Considering the Delta CHA_2_DS_2_-VASc score versus baseline score, the NRI was 14.4% (95% CI: 13.5 to 15.4%; *p* < 0.0001). Regarding the follow-up score vs the Delta score, NRI was 7.5% (95% CI: 6.5% to 8.4%; *p* < 0.0001). Using DCA, assuming that a high risk identified by one of the tests would result in different treatment, FU CHA_2_DS_2_-VASc score was superior to the “treat all” strategy and the “treat none” strategy. The FU CHA_2_DS_2_-VASc score was likewise superior to the CHA_2_DS_2_-VASc score and Delta CHA_2_DS_2_-VASc score for any threshold probability (Figure 3).

### 3.2. Slope of the Change in CHA_2_DS_2_-VASc Score

The slope was higher for patients with ischemic stroke than in those with no ischemic stroke (0.9 vs. 0.7; *p* < 0.0001), and this was also seen with different Delta CHA_2_DS_2_-VASc score (Table 4). The OR for suffering ischemic stroke was 1.29 (95% CI: 1.27 to 1.31; *p* < 0.0001) per 1 unit of the slope for patients with a Delta CHA_2_DS_2_-VASc score ≥1. This increased from 1.22 (95% CI: 1.19 to 1.24) for patients with a Delta CHA_2_DS_2_-VASc score = 1 to 1.64 (95% CI: 1.37 to 1.96) for patients with a Delta CHA_2_DS_2_-VASc score ≥4. The AUCs of the slope for prediction of ischemic stroke were marginally higher for patients with higher Delta CHA_2_DS_2_-VASc scores (2 and ≥3) than for those with lower Delta CHA_2_DS_2_-VASc score = 1 (Supplemental Figure S2).

## 4. Discussion

In this study from a white European nationwide AF cohort, our main findings are: (1) Stroke risk (assessed by CHA_2_DS_2_-VASc score) was non-static, and many patients developed ≥1 new stroke risk factor(s) before presenting with ischemic stroke; and (2) the follow-up CHA_2_DS_2_-VASc score and its evolution (i.e.,‘Delta CHA_2_DS_2_-VASc’, reflecting the modification in stroke risk profile between baseline and follow-up) were better predictors of ischemic stroke than relying on the baseline CHA_2_DS_2_-VASc score.

To our knowledge, this is the first report assessing the dynamic nature of stroke risk in a non-Asian cohort, using a large dataset from the large France nationwide administrative hospital-discharge database. The present study from France is the first from a non-Asian cohort, and was substantially larger than either the Taiwan and South Korea studies. In contrast to prior studies, we found that the follow-up CHA_2_DS_2_-VASc score had the best value for predicting ischemic stroke, as shown by AUCs and NRI.

In the study from Taiwan, Chao et al [3] reported that among AF patients with ischemic stroke, 89% had a Delta CHA_2_DS_2_-VASc score ≥1 whereas this was the case for only 55% in patients without ischemic stroke, and 64% patients had ≥1 new-onset comorbidity, the most common being hypertension. The Delta CHA_2_DS_2_-VASc score was a significant predictor of ischemic stroke performing better than baseline or follow-up CHA_2_DS_2_-VASc scores.

By contrast to the follow-up CHA_2_DS_2_-VASc score, an increasing Delta CHA_2_DS_2_-VASc score above 1 (2, 3, and ≥4) in our study was not significantly associated with a higher risk of ischemic stroke. This may seem counterintuitive and different to Chao et al [3], but our patients with highest Delta CHA_2_DS_2_-VASc in our study had longer follow-up, lower prevalence of those age above 75 and lower baseline CHA_2_DS_2_-VASc score. This may partly explain why the univariate analysis by strata of the Delta CHA_2_DS_2_-VASc score did not find increasing risk when Delta CHA_2_DS_2_-VASc score increased above 2. Of note, the predictive value of the slope of CHA_2_DS_2_-VASc score change in the different strata confirms that the increasing number of risk factors per year provides consistent evidence on the increasing risk of stroke in our population.

In a similar analysis in Korea, studying more than 160,000 AF patients with no anticoagulation, Yoon et al [4] reported that during follow-up of 10 years, the rate of ischemic stroke was higher when patients had incident risk factors, and were re-classified into categories with higher CHA_2_DS_2_-VASc score. Thus, the two Asian study and our results reaffirm why assessment of stroke risk is needed at each contact with the patient, as addition of risk factors and increasing CHA_2_DS_2_-VASc score results over time in a greater stroke risks. Of note, the dynamic nature of risk is not only evident for stroke risk, but also for bleeding risk. Chao et al also showed that the best predictive value for major bleeding was the follow-up and change HAS-BLED score [9,10].

Assessment of clinical usefulness (using Decision Curve Analysis) re-emphasizes the value of follow-up stroke risk assessment. When assessing the slope of change in CHA_2_DS_2_-VASc scores, the predictive value was better for patients when they had higher Delta CHA_2_DS_2_-VASc scores (2 and ≥3) compared to those who had a lower Delta CHA_2_DS_2_-VASc score of 1. Indeed, the “take home” message should be that clinicians should not rely on baseline ’one off’ stroke risk assessment, but regular re-assessment, for the follow-up or change in stroke risk profile, which is a better predictor compared to the baseline assessment.

### Limitations

One limitation of this study was related to its observational and retrospective nature. This analysis is limited by its registry design, and errors for classification or coding may be present. Identifying AF and comorbidities is challenging. However, ICD-10 is considered reliable for identifying AF, stroke and stroke risk factors [11,12,13]. The PMSI was previously verified and used for epidemiologic purpose in AF patients or those with ischemic stroke [7]. We were not able to differentiate paroxysmal AF from persistent and permanent AF, whereas patients with permanent AF may have a higher risk of stroke. Events included were only in-hospital, and we had no outpatient data. Results apply to inpatients with AF and may not apply to all AF patients. Data were based on the diagnostic codes registered for reimbursement purposes and were not externally checked with a potential information bias. However, our study aimed to provide a simple clinical approach and a global picture at a national level not limited to tertiary referral centers. Drug therapies, particularly oral anticoagulation use, were not included in the analysis, which may impact on event rates at follow-up. The recent EORP-AF registry indicates that the rate of anticoagulation therapy based on CHA_2_DS_2_VASc score (≥2) in Europe is generally above 85% [14] and this should apply to our nationwide analysis. Another limitation is the lack of available information concerning time in therapeutic range for international normalized ratios for patients taking vitamin K antagonist. However, the study size of the population including every hospitalization in France with a negligible risk of follow-up loss may compensate for some of these limitations.

## 5. Conclusions

Stroke risk (CHA_2_DS_2_-VASc score) was dynamic, and many AF patients had ≥1 new stroke risk factor(s) before they suffered ischemic stroke. The follow-up CHA_2_DS_2_-VASc score and its change (i.e., Delta CHA_2_DS_2_-VASc, picturing the evolution in stroke risk profile between baseline and follow-up) were better predictors of ischemic stroke than relying on the baseline CHA_2_DS_2_-VASc score. This emphasizes that stroke risk in AF is an evolving process when age increases and when new comorbidities appear, and regular re-assessment of risk is needed.

## Figures and Tables

**Table 1:** Baseline characteristics of AF patients.

	Total	No Ischemic Stroke	Ischemic Stroke	*p*
	(*n* = 608108)	(*n* = 588026)	(*n* = 20082)	
Characteristics at baseline				
Age, years	72.7 ± 14.6	72.5 ± 14.6	78.3 ± 10.8	<0.0001
Age 65–74 yrs	135994 (22.4)	132380 (22.5)	3614 (18.0)	<0.0001
Age ≥75 yrs	313332 (51.5)	299213 (50.9)	14119 (70.3)	<0.0001
Gender (male)	341299 (56.1)	331608 (56.4)	9691 (48.3)	<0.0001
CHA_2_DS_2_-VASc score	1.7 ± 1.1	1.7 ± 1.1	2.1 ± 0.9	<0.0001
CHA_2_DS_2_-VASc score = 0	110373 (18.2)	108728 (18.5)	1645 (8.2)	<0.0001
CHA_2_DS_2_-VASc score = 1	131674 (21.7)	128849 (21.9)	2825 (14.1)	<0.0001
CHA_2_DS_2_-VASc score = 2	197673 (32.5)	190410 (32.4)	7263 (36.2)	<0.0001
CHA_2_DS_2_-VASc score = 3	168388 (27.7)	160039 (27.2)	8349 (41.6)	<0.0001
Coronary artery disease	52807 (8.7)	50744 (8.6)	2063 (10.3)	<0.0001
Previous pacemaker or Defibrillator	8317 (1.4)	8022 (1.4)	295 (1.5)	0.21
Smoker	26202 (4.3)	25687 (4.4)	515 (2.6)	<0.0001
Dyslipidemia	38136 (6.3)	36967 (6.3)	1169 (5.8)	0.01
Obesity	33410 (5.5)	32700 (5.6)	710 (3.5)	<0.0001
Alcohol related diagnoses	24514 (4.0)	23803 (4.0)	711 (3.5)	0.0003
Abnormal renal function	10898 (1.8)	10555 (1.8)	343 (1.7)	0.36
Lung disease	60242 (9.9)	58721 (10.0)	1521 (7.6)	<0.0001
Liver disease	13894 (2.3)	13572 (2.3)	322 (1.6)	<0.0001
Thyroid diseases	39551 (6.5)	38244 (6.5)	1307 (6.5)	0.98
Inflammatory disease	22307 (3.7)	21587 (3.7)	720 (3.6)	0.52
Anemia	54504 (9.0)	52925 (9.0)	1579 (7.9)	<0.0001
Previous cancer	93633 (15.4)	91466 (15.6)	2167 (10.8)	<0.0001
Characteristics after the follow-up				
Age, years	74.8 ± 14.6	74.6 ± 14.7	80.8 ± 10.8	<0.0001
Age 65–74 yrs	128281 (21.1)	125254 (21.3)	3027 (15.1)	<0.0001
Age ≥75 yrs	343328 (56.5)	328135 (55.8)	15193 (75.7)	<0.0001
CHA_2_DS_2_-VASc score	2.4 ± 1.5	2.3 ± 1.5	3.2 ± 1.3	<0.0001
CHA_2_DS_2_-VASc score = 0	76845 (12.6)	76186 (13.0)	659 (3.3)	<0.0001
CHA_2_DS_2_-VASc score = 1	100752 (16.6)	99369 (16.9)	1383 (6.9)	<0.0001
CHA_2_DS_2_-VASc score = 2	144335 (23.7)	140771 (23.9)	3564 (17.7)	<0.0001
CHA_2_DS_2_-VASc score = 3	160270 (26.4)	154124 (26.2)	6146 (30.6)	<0.0001
CHA_2_DS_2_-VASc score = 4	78774 (13.0)	73630 (12.5)	5144 (25.6)	<0.0001
CHA_2_DS_2_-VASc score = 5	37770 (6.2)	35291 (6.0)	2479 (12.3)	<0.0001
CHA_2_DS_2_-VASc score = 6	8584 (1.4)	7941 (1.4)	643 (3.2)	<0.0001
CHA_2_DS_2_-VASc score = 7	778 (0.1)	714 (0.1)	64 (0.3)	<0.0001
New-onset comorbidities				
Hypertension	156081 (25.7)	146131 (24.9)	9950 (49.5)	<0.0001
Heart failure	125684 (20.7)	119795 (20.4)	5889 (29.3)	<0.0001
Diabetes mellitus	27105 (4.5)	25531 (4.3)	1574 (7.8)	<0.0001
Vascular disease	40512 (6.7)	37503 (6.4)	3009 (15.0)	<0.0001
Any new-onset comorbidity	227993 (37.5)	214952 (36.6)	13041 (64.9)	<0.0001
Delta CHA_2_DS_2_-VASc score	0.7 ± 0.9	0.6 ± 0.9	1.1 ± 1.0	<0.0001
Delta CHA_2_DS_2_-VASc score = 0	357349 (58.8)	350738 (59.6)	6611 (32.9)	<0.0001
Delta CHA_2_DS_2_-VASc score = 1	140768 (23.1)	133503 (22.7)	7265 (36.2)	<0.0001
Delta CHA_2_DS_2_-VASc score = 2	76373 (12.6)	72090 (12.3)	4283 (21.3)	<0.0001
Delta CHA_2_DS_2_-VASc score = 3	27067 (4.5)	25498 (4.3)	1569 (7.8)	<0.0001
Delta CHA_2_DS_2_-VASc score = 4	5809 (1.0)	5484 (0.9)	325 (1.6)	<0.0001
Delta CHA_2_DS_2_-VASc score = 5	742 (0.1)	713 (0.1)	29 (0.1)	0.36

Values are mean ± SD or *n* (%). AF = atrial fibrillation; CHA_2_DS_2_-VASc = congestive heart failure, hypertension, age ≥75 years, diabetes mellitus, prior stroke or transient ischemic attack, vascular disease, age 65–74 years, sex category (female).

**Table 2:** Characteristics of patients with AF stratified by delta CHA_2_DS_2_-VASc score.

	Delta CHA_2_DS_2_-VASc score = 0	Delta CHA_2_DS_2_-VASc score = 1	Delta CHA_2_DS_2_-VASc score = 2	Delta CHA_2_DS_2_-VASc score ≥3
	(*n* = 357349)	(*n* = 140768)	(*n* = 76373)	(*n* = 33618)
*Characteristics at baseline*				
Age, years	70.7 ± 16.1	75.1 ± 12.2	76.7 ± 10.8	74.6 ± 9.6
Age 65–74 yrs	75426 (21.1)	31574 (22.4)	17578 (23.0)	11416 (34.0)
Age ≥75 yrs	171231 (47.9)	79587 (56.5)	46575 (61.0)	15939 (47.4)
Gender (male)	200713 (56.2)	77384 (55.0)	41750 (54.7)	21452 (63.8)
CHA_2_DS_2_-VASc score	1.6 ± 1.1	1.8 ± 1.0	1.9 ± 1.0	1.6 ± 1.0
CHA_2_DS_2_-VASc score = 0	76841 (21.5)	20344 (14.5)	8444 (11.1)	4607 (13.7)
CHA_2_DS_2_-VASc score = 1	79937 (22.4)	28338 (20.1)	14311 (18.7)	9081 (27.0)
CHA_2_DS_2_-VASc score = 2	107111 (30.0)	49157 (34.9)	28689 (37.6)	12829 (38.2)
CHA_2_DS_2_-VASc score = 3	93460 (26.2)	42929 (30.5)	24929 (32.6)	7101 (21.1)
Coronary artery disease	18290 (5.1)	17106 (12.2)	10545 (13.8)	6866 (20.4)
Previous pacemaker or Defibrillator	4331 (1.2)	2237 (1.6)	1244 (1.6)	505 (1.5)
Smoker	17859 (5.0)	5074 (3.6)	2231 (2.9)	1038 (3.1)
Dyslipidemia	21218 (5.9)	9811 (7.0)	4929 (6.5)	2178 (6.5)
Obesity	19348 (5.4)	7493 (5.3)	4323 (5.7)	2246 (6.7)
Alcohol related diagnoses	15704 (4.4)	5050 (3.6)	2492 (3.3)	1268 (3.8)
Abnormal renal function	5846 (1.6)	2733 (1.9)	1585 (2.1)	734 (2.2)
Lung disease	35331 (9.9)	13682 (9.7)	7692 (10.1)	3537 (10.5)
Liver disease	8938 (2.5)	2956 (2.1)	1407 (1.8)	593 (1.8)
Thyroid diseases	23446 (6.6)	9591 (6.8)	4866 (6.4)	1648 (4.9)
Inflammatory disease	13299 (3.7)	5104 (3.6)	2773 (3.6)	1131 (3.4)
Anemia	34058 (9.5)	12223 (8.7)	6011 (7.9)	2212 (6.6)
Previous cancer	61422 (17.2)	20163 (14.3)	8798 (11.5)	3250 (9.7)
*Characteristics after the follow-up*				
Follow-up duration (years)	1.2 ± 1.9	3.0 ± 2.4	3.8 ± 2.4	4.8 ± 2.3
Age, years	71.9 ± 15.9	78.1 ± 11.9	80.5 ± 10.4	79.3 ± 9.2
Age 65–74 yrs	75426 (21.1)	31250 (22.2)	14225 (18.6)	7380 (22.0)
Age ≥75 yrs	171231 (47.9)	91132 (64.7)	56420 (73.9)	24545 (73.0)
CHA_2_DS_2_-VASc score	1.6 ± 1.1	2.8 ± 1.0	3.9±1.0	4.9 ± 1.0
CHA_2_DS_2_-VASc score = 0	76845 (21.5)	0 (0.0)	0 (0.0)	0 (0.0)
CHA_2_DS_2_-VASc score = 1	79940 (22.4)	20812 (14.8)	0 (0.0)	0 (0.0)
CHA_2_DS_2_-VASc score = 2	107108 (30.0)	28419 (20.2)	8808 (11.5)	0 (0.0)
CHA_2_DS_2_-VASc score = 3	93456 (26.2)	48898 (34.7)	14432 (18.9)	3484 (10.4)
CHA_2_DS_2_-VASc score = 4	0 (0.0)	42639 (30.3)	28480 (37.3)	7655 (22.8)
CHA_2_DS_2_-VASc score = 5	0 (0.0)	0 (0.0)	24653 (32.3)	13117 (39.0)
CHA_2_DS_2_-VASc score = 6	0 (0.0)	0 (0.0)	0 (0.0)	8584 (25.5)
CHA_2_DS_2_-VASc score = 7	0 (0.0)	0 (0.0)	0 (0.0)	778 (2.3)
Hypertension	0 (0.0)	61486 (43.7)	62627 (82.0)	31968 (95.1)
Heart failure	0 (0.0)	45143 (32.1)	51028 (66.8)	29513 (87.8)
Diabetes mellitus	0 (0.0)	4590 (3.3)	9115 (11.9)	13400 (39.9)
Vascular disease	0 (0.0)	6783 (4.8)	13639 (17.9)	20090 (59.8)
Any new-onset comorbidity	0 (0.0)	118002 (83.8)	76373 (100.0)	33618 (100.0)

Values are mean ± SD or *n* (%). Abbreviation as in Table 1. IQR = interquartile range.

**Table 3:** Risk of ischemic stroke in AF patients with different baseline follow-up and delta CHA_2_DS_2_-VASc score (mean (SD) 2.2 (2.4), median (IQR) 1.2 (0.1–3.8) years).

	Number of Patients	Number of Incident Ischemic Stroke	Incidence of Ischemic Stroke (%/year)	Hazard. Ratio (95%CI)	*p*
**Baseline CHA_2_DS_2_-VASc score**					
0 (reference group)	111,269	1687	0.67	1.00	
1	131,937	2852	0.91	1.35 (1.27–1.43)	<0.0001
2	197,176	7238	1.69	2.54 (2.41–2.68)	<0.0001
3	167,726	8305	2.52	3.80 (3.61–4.01)	<0.0001
					
**Follow-up CHA_2_DS_2_-VASc score**					
0 (reference group)	76,845	659	0.60	1.00	
1	100,752	1383	0.77	1.30 (1.18–1.42)	<0.0001
2	144,335	3564	1.34	2.25 (2.07–2.44)	<0.0001
3	160,270	6146	1.91	3.20 (2.95–3.47)	<0.0001
4	78,774	5144	1.98	3.38 (3.12–3.67)	<0.0001
≥5	47,132	3186	1.70	2.90 (2.66–3.15)	<0.0001
					
**Delta CHA_2_DS_2_-VASc score**					
0 (reference group)	357,349	6611	1.50	1.00	
1	140,768	7265	1.69	1.16 (1.12–1.20)	<0.0001
2	76,373	4283	1.46	0.99 (0.96–1.03)	0.75
3	27,067	1569	1.27	0.85 (0.81–0.90)	<0.0001
≥4	6551	354	0.99	0.65 (0.58–0.72)	<0.0001

Values are *n* (incidence rate, %/year); CI = confidence interval. AF = atrial fibrillation; SD = standard deviation; IQR = interquartile range.

**Table 4:** Slope of the change of c CHA_2_DS_2_-VASc score and the risk of ischemic stroke in each group of delta CHA_2_DS_2_-VASc score.

	No Ischemic Stroke	Ischemic Stroke	*p* value	Odds Ratio (95% CI) of Ischemic Stroke Per Unit Slope Change	*p* value
	(*n* = 237288)	(*n* = 13471)			
**Delta CHA_2_DS_2_-VASc Score**					
**All (≥1)**	0.7 ± 0.8	0.9 ± 1.2	<0.0001	1.29 (1.27–1.31)	<0.0001
**1**	0.7 ± 0.9	0.9 ± 1.3	<0.0001	1.22 (1.19–1.24)	<0.0001
**2**	0.7 ± 0.6	0.9 ± 1.2	<0.0001	1.48 (1.44–1.53)	<0.0001
**3**	0.7 ± 0.5	0.9 ± 0.9	<0.0001	1.54 (1.44–1.64)	<0.0001
**≥ 4**	0.6 ± 0.4	0.8 ± 0.8	<0.0001	1.64 (1.37–1.96)	<0.0001

## Supplementary Materials

The following are available online at https://www.mdpi.com/2077-0383/9/4/1234/s1, Supplemental Figure S1. Number of new-onset comorbidities for patients who experienced ischemic stroke. Supplemental Figure S2. AUCs for the slopes in predicting ischemic stroke in different Delta CHA_2_DS_2_-VASc scores.
