# Supplementary material for: Incident Comorbidities, Aging and the Risk of Stroke in 608,108 Patients with Atrial Fibrillation: A Nationwide Analysis
Source: J Clin Med. 2020 Apr 24;9(4):1234. doi: 10.3390/jcm9041234 (PMC7230460; doi:10.3390/jcm9041234)
Supplement: Supplementary file 1 [file jcm-09-01234-s001.pdf]

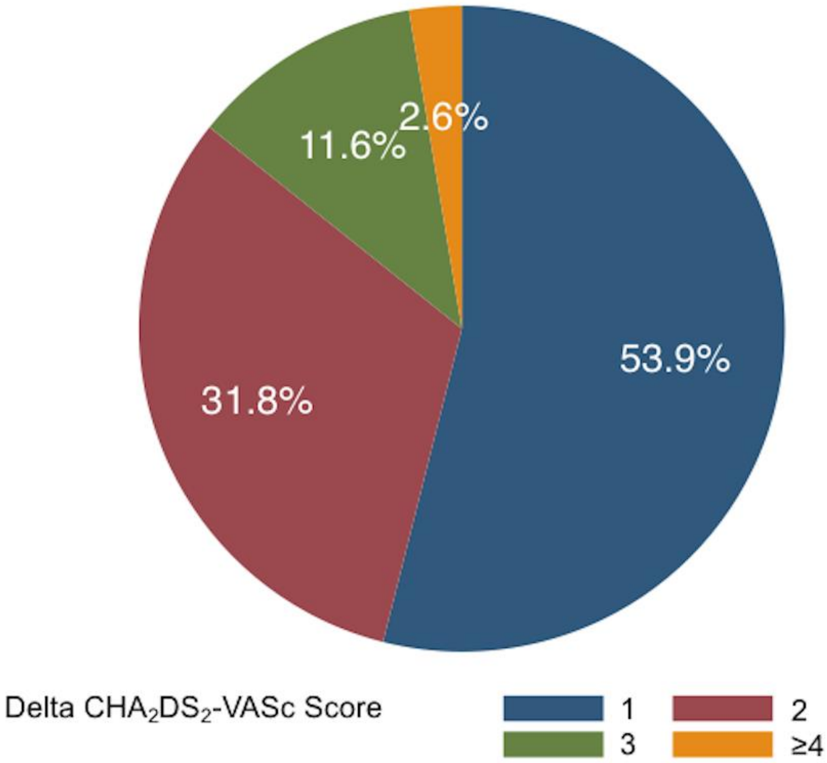

Supplemental Figure 1. Number of new-onset comorbidities for patients who experienced ischemic stroke.

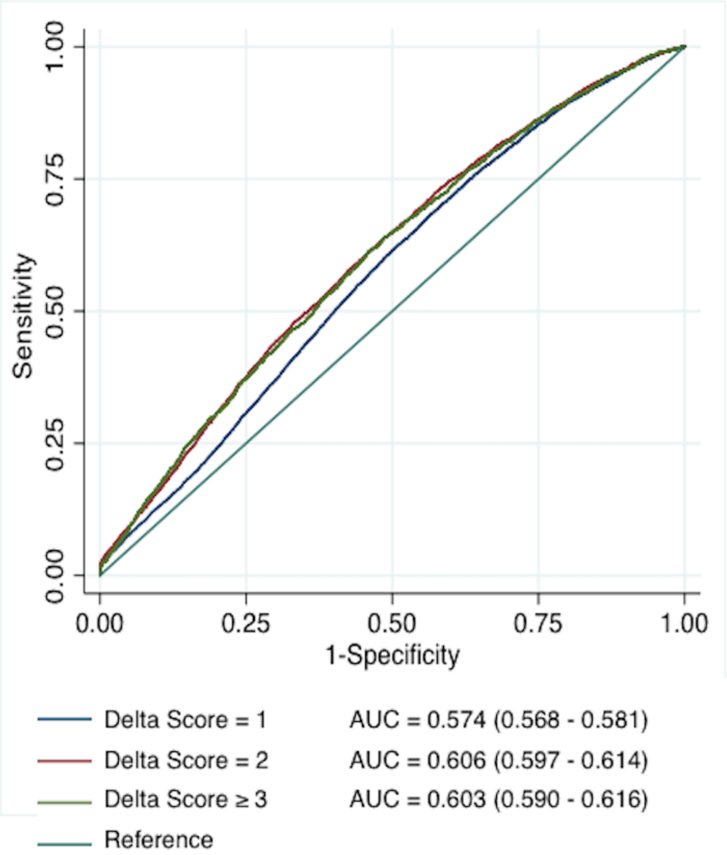

Supplemental Figure 2. AUCs for the slopes in predicting ischemic stroke in different Delta CHA_2DS_2-VASc scores
